# ﻿ *Myrsinecirrhosa* (Primulaceae), a distinctive new shrub species from Kaua‘i, Hawaiian Islands

**DOI:** 10.3897/phytokeys.243.123694

**Published:** 2024-06-19

**Authors:** David H. Lorence, Kenneth R. Wood, Marc S. Appelhans, Warren L. Wagner

**Affiliations:** 1 National Tropical Botanical Garden, 3530 Papalina Road, Kalāheo, HI 96741, USA National Tropical Botanical Garden Kalāheo United States of America; 2 Department of Systematics, Biodiversity and Evolution of Plants, Albrecht-von-Haller Institute of Plant Sciences, University of Goettingen, Untere Karspuele 2, 37073 Goettingen, Germany University of Goettingen Goettingen Germany; 3 Department of Botany, National Museum of Natural History, Smithsonian Institution, P.O. Box 37012, MRC 166, Washington, DC 20013-7012, USA Department of Botany, National Museum of Natural History Washington United States of America

**Keywords:** Conservation, Hawaiian Islands, Kaua‘i endemism, *Myrsine*, Primulaceae

## Abstract

*Myrsinecirrhosa* Lorence & K.R.Wood (Primulaceae), a new single-island endemic shrub species from Kaua‘i, Hawaiian Islands, is described and illustrated. Notes on its distribution, ecology and conservation status are included. The new species is known from an area with ca. 45 individuals, where it is restricted to the remote central windward region of Kaua‘i in open bogs and along open windy ridges. Suggested IUCN Red List status is CR (Critically Endangered). It differs from its Kaua‘i congeners by its longer petals and narrowly elliptic leaves with strongly undulate margins and tendril-like apex. Phylogenetic analysis using RADseq data supports the recognition of this new species.

## ﻿Introduction

*Myrsine* L., in the family Primulaceae, is a pantropical to subtropical genus comprising ca. 200 species of shrubs and trees occurring throughout Africa, Asia and most of the Pacific Basin (Appelhans et al. 2020; Lorence and Wagner 2020). Although *Rapanea* Aubl. and *Suttonia* A.Rich. were previously recognised as distinct genera, both morphological (Hosaka 1940) and molecular phylogenetic studies (Appelhans et al. 2020) confirm they are nested within a monophyletic *Myrsine*. The genus is taxonomically complex, certain species are morphologically variable and the differences between species are mostly vegetative and often subtle (Wagner et al. 1999; Lorence and Wagner 2020).

*Myrsine* species range from small shrubs to medium-sized trees reaching 8 m tall or more, with simple, alternate leaves punctate with secretory canals. Inflorescences are in fascicles, umbels or glomerules produced along the branches on short woody knobs (spurs), either axillary or often below the leaves. Flowers are either perfect or unisexual (and then the plants dioecious) and fruits are subglobose, 1-seeded drupes (Wagner et al. 1999). In the Hawaiian Islands, species of *Myrsine* are associated with insect pollination and seed dispersal by forest birds (Sakai et al. 1995).

Hosaka (1940) wrote a revision of the Hawaiian *Myrsine* and recognised 25 species, based on morphological characters, whereas Wagner et al. (1999) recognised 20 species. Currently there are 19 recognised species in the Hawaiian Islands, with *M.emarginata* (Rock) Hosaka now being a synonym of *M.lessertiana* A.DC. (Wagner et al. 2023; POWO 2024). The Hawaiian group was resolved as monophyletic, based on their ITS/ETS phylogeny (Appelhans et al. 2020) ranking it amongst the ten largest Hawaiian plant radiations. The greatest species diversity occurs on Kaua‘i, the oldest of the main Hawaiian Islands at 4.7 mya (Price and Clague 2002). During the course of fieldwork on high summit regions of Kaua‘i, a distinctive new species of *Myrsine* was collected at three localities; it is described below and subsequently referred to as *Myrsinecirrhosa*. This new species is most similar morphologically to *M.helleri* (O.Deg. & I.Deg.) H.St.John and *M.fosbergii* Hosaka from which it differs by its longer petals and leaves with a combination of strongly undulate margins and tendril-like, cirrhose apices. With the inclusion of this new species, Kaua‘i now harbours 15 species, of which 12 are single island endemics. Unfortunately, material of the new species was not available for study by Appelhans et al. (2020), who studied Hawaiian *Myrsine* using RADseq. We, therefore, sequenced two specimens of the new species plus additional samples of Kaua‘i species in the framework of this project in order to evaluate its phylogenetic position.

## ﻿Methods

### ﻿RADseq: Taxon sampling

This study is largely based on Appelhans et al. (2020). In addition to that dataset, we added two samples of the new species collected from spatially separated individuals in the same (Wai‘ahi) population, as well as one sample each of *M.fosbergii*, *M.helleri* and *M.linearifolia* Hosaka (Suppl. material 5). With the exception of the O‘ahu endemic *M.degeneri* Hosaka, all currently accepted species of Hawaiian *Myrsine* have now been included in a phylogenomic study based on RADseq. Appelhans et al. (2020) used two species of *Ardisia* Sw. as outgroups. In order to have more closely-related taxa as outgroups, we omitted the *Ardisia* samples and rooted the phylogenetic trees with the earliest branching clade of Hawaiian *Myrsine* that consists of *M.lanaiensis* Hillebr. and two specimens of the polyphyletic *M.lessertiana* (Clade C in Appelhans et al. 2020). The final dataset included 31 samples (Suppl. material 5).

### ﻿RADseq: DNA Extraction, Library Preparation and Sequencing

Genomic DNA was extracted from silica-dried leaf material using the Qiagen DNeasy Plant Mini Kit (Qiagen, Hilden, Germany) following the manufacturer’s instructions. A Qubit 3.0 fluorometer (Thermo Fisher Scientific, Waltham, MA, USA) in combination with the Qubit dsDNA Broad Range assay kit was used to measure the quality and quantity of the DNA extractions. The normalised samples (30 ng/µl) were sent to Floragenex (Eugene, OR, USA) for library preparation using the restriction enzyme *Sfb*I and for sequencing on an Illumina HiSeq machine, which produced 2 × 150 bp paired-end reads. De-multiplexed raw reads for all samples have been deposited at the Sequence Read Archive (SRA; https://www.ncbi.nlm.nih.gov/sra; Suppl. material 5) under the BioProject number PRJNA614459 together with the sequence reads from Appelhans et al. (2020).

### ﻿RADseq: Bioinformatics and Phylogenetic Reconstruction

All steps, from demultiplexing to the calculation of the alignments of RAD loci, were carried out using ipyrad 0.9.52 (Eaton and Overcast 2020). One mismatch in the barcode sequence was allowed for demultiplexing. Adapters were removed and read filtering was done by deleting reads with more than five low-quality bases (< 20), a phred Q score offset of 33 and removing trimmed reads shorter than 35 bp. RAD loci were assembled *de novo* using clustering thresholds of 85% for within and amongst sample clustering. Appelhans et al. (2020) tested two different clustering thresholds (85% and 90%) and did not record any significant differences, so that only the 85% threshold – which is the default setting – is used here. As a trade-off between number of RAD loci included and missing data, five datasets were assembled that differed in the minimum number of samples recovered per RAD locus. This minimum number was set to either > 25% (min8; a RAD locus was only included in the alignment in case a sequence had been recovered for at least eight of the 31 samples), > 33% (min11), > 50% (min16), > 66% (min21) or > 75% (min24). With increasing “min” numbers, the numbers of RAD loci, but also the amount of missing data, were expected to drop. The resulting alignments were used for phylogenetic reconstruction using RAxML 8.2.4. (Stamatakis 2014), applying the GTR + Γ model and calculating 100 bootstrap replicates.

All ipyrad and RAxML analyses were computed on the high-performance computing cluster of the “*Gesellschaft für wissenschaftliche Datenverarbeitung Göttingen*” (GWDG), Germany (https://gwdg.de/en/hpc/services/).

### ﻿Morphological analyses

Herbarium specimens of the new species have been deposited at PTBG and other herbaria listed under specimens examined and in Suppl. material 5 (acronyms according to Thiers (2018)). All measurements were taken from dried herbarium specimens and field notes and are presented in the descriptions as follows: length × width, followed by units of measurement (m, mm or cm). The authors have examined all specimens cited. We assessed the extinction risk for the new species following the IUCN Red List Categories and Criteria (IUCN 2012) and guidelines of the IUCN Standards and Petitions Committee (IUCN 2022). The extent of occurrence and area of occupancy were calculated by using ArcMap 10.6.1 (ESRI 2011) in relation to coordinates recorded while collecting herbarium specimens and making field observations. The coordinates latitude and longitude have been truncated to protect the exact holotype location from unauthorised access.

## ﻿Results and discussion

### ﻿Sequencing and RADseq datasets

The sequencing runs yielded an average of 4,425,834 raw reads per sample of which an average of 4,422,490 reads remained after trimming. This is less compared to the sequence reads from the Appelhans et al. (2020) study (5,505,232 raw reads, 5,281,476 reads after filtering; Suppl. material 5), but the newly-generated sequences had more bp overall because they were sequenced with the 2 × 150 bp paired-end chemistry instead of single end 100 bp in the previous study. Despite the higher number of bp in the newly-sequenced samples, the numbers of retained RAD loci was much lower for the new samples (18× lower in the min8 dataset; 19× lower in min11; 16× lower in min16; 5× lower in min21; 2× in min24 dataset), which might be due to the different sequencing strategies (2 × 150 bp vs. 1 × 100 bp) and the different fragment size selection during library preparation in particular.

As expected, the assembled datasets varied greatly in numbers of RAD loci, alignment length and amount of missing data. The min8 dataset contained 55,048 loci with a concatenated alignment length of 4,832,837 bp and 49.87% missing data, while the min24 dataset contained only 317 loci with an alignment length of 35,821 bp and 35.78% missing data (Suppl. material 5).

### ﻿Phylogeny

**Figure 1.**
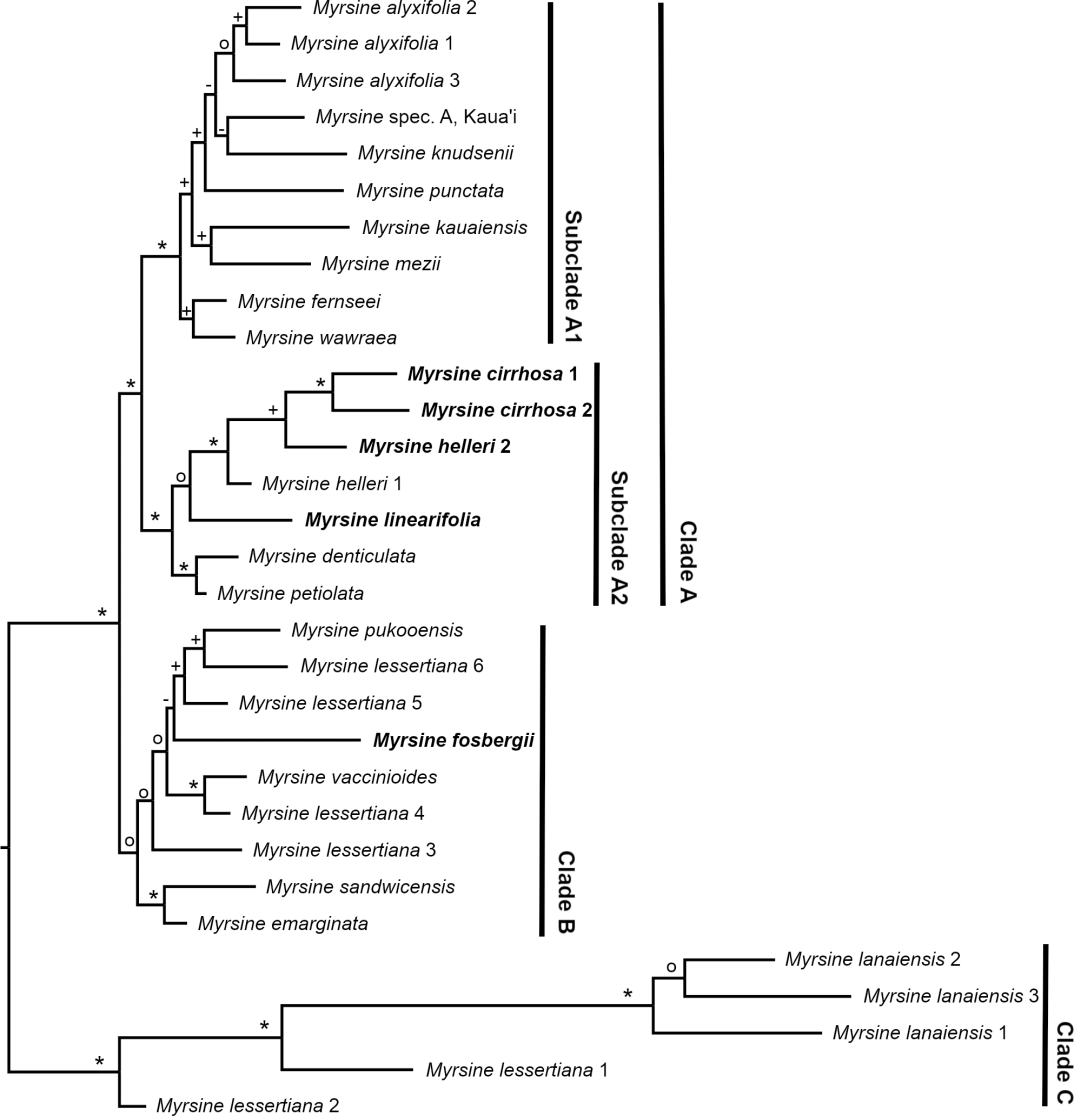
RADseq phylogeny of Hawaiian *Myrsine* based on the min16 dataset. Samples with newly-generated sequence data are highlighted in bold. Symbols at branches represent bootstrap support (bs) values (*: maximum bs; +: bs of 90 or higher; °: bs of 70 or higher; -: bs < 70).

Despite the large differences in the datasets, the consensus trees showed a congruent backbone, in which the same three main clades (Clades A, B and C) and the division of Clade A in two subclades (Subclades A1 and A2) are inferred as in Appelhans et al. (2020) (Fig. 1, Suppl. materials 1–4). However, average branch support differs amongst the datasets. The min16 and min8 consensus trees had the highest average branch support of 89.2% bs and 88.9% bs, respectively, followed by the min11 consensus tree with 84.2% bp, the min21 consensus tree with 79.9% bs and the min24 consensus tree with only 60.0% bs. Due to the higher support values, we discuss the consensus tree, based on the min16 dataset (Fig. 1, Suppl. materials 1–4) and the other consensus trees are mentioned in case of supported differences.

The two samples of the new species *M.cirrhosa* and the specimens of *M.helleri* and *M.linearifolia* are resolved within Subclade A2 (Fig. 1). This subclade consists of species endemic to Kaua‘i. With the exception of *M.denticulata* (Wawra) Hosaka, which has small leaves with a dentate margin, all species in this subclade are characterised by linear, narrowly elliptic to narrowly lanceolate leaves (Wagner et al. 1999; Appelhans et al. 2020). The new species *M.cirrhosa* fits well into this clade regarding its distribution and morphology.

*Myrsinecirrhosa* is resolved as the closest relative of *M.helleri*. In the min21 and min24 phylogenies, the two species are resolved as monophyletic sister species (Suppl. materials 3, 4). The other phylogenies did not resolve *M.helleri* as monophyletic. In the min11 and min16 phylogenies, *M.helleri* forms a grade at the base of *M.cirrhosa* (Suppl. material 2, Fig. 1). In the min8 phylogeny, one sample of *M.helleri* is sister to a clade that consists of the second *M.helleri* sample as well as *M.cirrhosa*, *M.fosbergii* and *M.linearifolia* (Suppl. material 1). A denser taxon sampling is needed to address the correct placement and potential polyphyly of *M.helleri*.

*Myrsinefosbergii* (Kaua‘i, O‘ahu) is part of Clade B in all analyses, except the phylogeny of the min8 dataset (Suppl. material 1), where it also belongs to subclade A2. In all, but the min8 phylogeny, the backbone support of Clade B is rather low, which is potentially caused by the *M.fosbergii* specimen. In the min8 phylogeny, bootstrap support values of Clade B are generally higher and also the placement of *M.fosbergii* in Subclade A2 as sister to *M.linearifolia* is highly supported. The placement of *M.fosbergii* in Subclade A2 is highly supported by morphology and the species is characterised by narrowly elliptic leaves. The different phylogenetic placements of this species might be due to the low number of recovered RAD loci instead of a biological reason such as introgression or hybridisation. The number of loci is the second lowest after *M.lanaiensis* 3 for the min8, min11, min16 and min21 datasets and is the third lowest after *M.lanaiensis* 3 and *M.knudsenii* (Rock) Hosaka for the min24 dataset (Suppl. material 5). On the other hand, the estimated heterozygosity, which is indicative of introgression and hybridisation, in the *M.fosbergii* sample is only slightly above the average (Suppl. material 5).

### ﻿Taxonomic treatment

#### ﻿ Myrsine cirrhosa

Taxon classificationPlantaeEricalesPrimulaceae

Lorence & K.R.Wood sp. nov.

9326BFA3-597F-5B19-87B7-5D966111C5CE

urn:lsid:ipni.org:names:77343738-1

Figs 2 , 3A, B

##### Diagnosis.

*Myrsinecirrhosa* is most similar morphologically to both *M.helleri* and *M.fosbergii*, from which it differs by its longer petals and leaves with a combination of strongly undulate margins and tendril-like, cirrhose apex.

**Figure 2.**
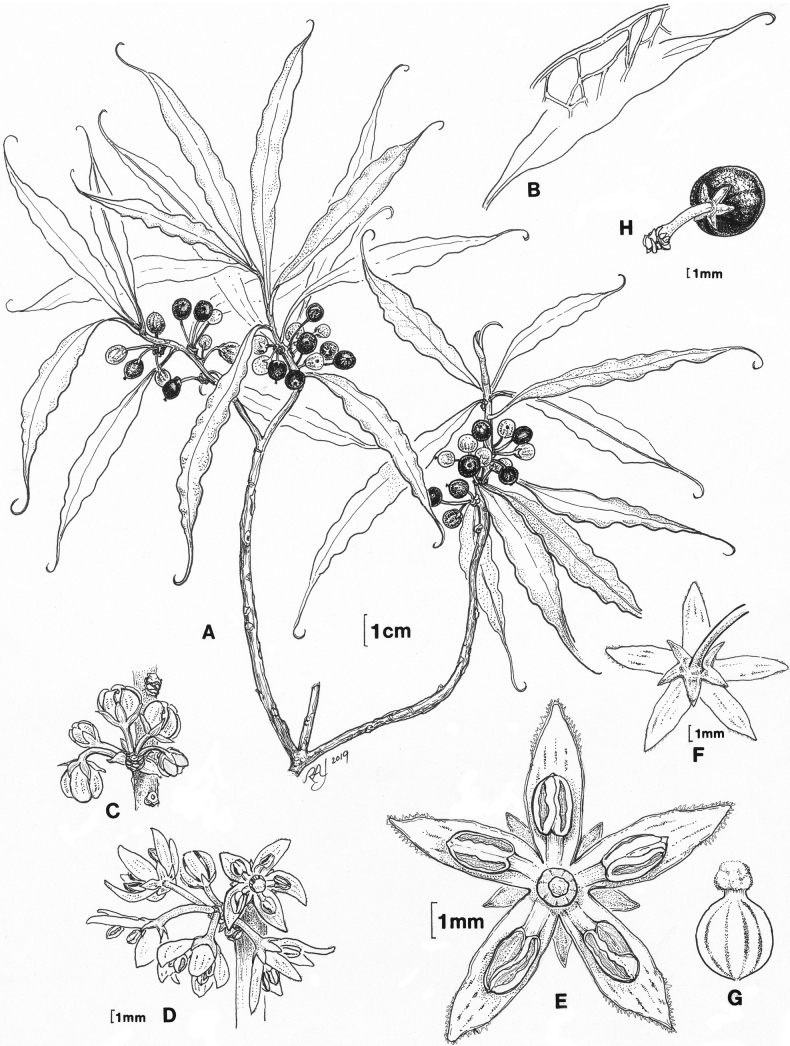
*Myrsinecirrhosa* Lorence & K.R.Wood **A** habit, fruiting branch **B** leaf showing cirrhose apex and detail of intramarginal venation **C** inflorescence in bud **D** inflorescence, flowers at anthesis **E** flower at anthesis, view from apex **F** flower at anthesis, view from base **G** pistil **H** mature drupe. **A, B** drawn from *Wood et al. 835* (PTBG), **C** drawn from *Wood & Query 12824* (PTBG), **D–G** drawn from *Wood et al.18139* (PTBG), **H** drawn from *Perlman & Wood 12747* (PTBG). Illustration by Robin Jess.

##### Type.

**USA. Hawaiian Islands: Kaua‘i**: Līhu‘e District, Kapalaoa, peak north of Wahiawa drainage, 21.99 N; -159.50 W, 930 m elev., 15 May 1991, *K. R. Wood et al. 835*, (holotype: PTBG-barcode 1000096825; isotypes (to be distributed): BISH, MO, NY, UC, US).

**Figure 3.**
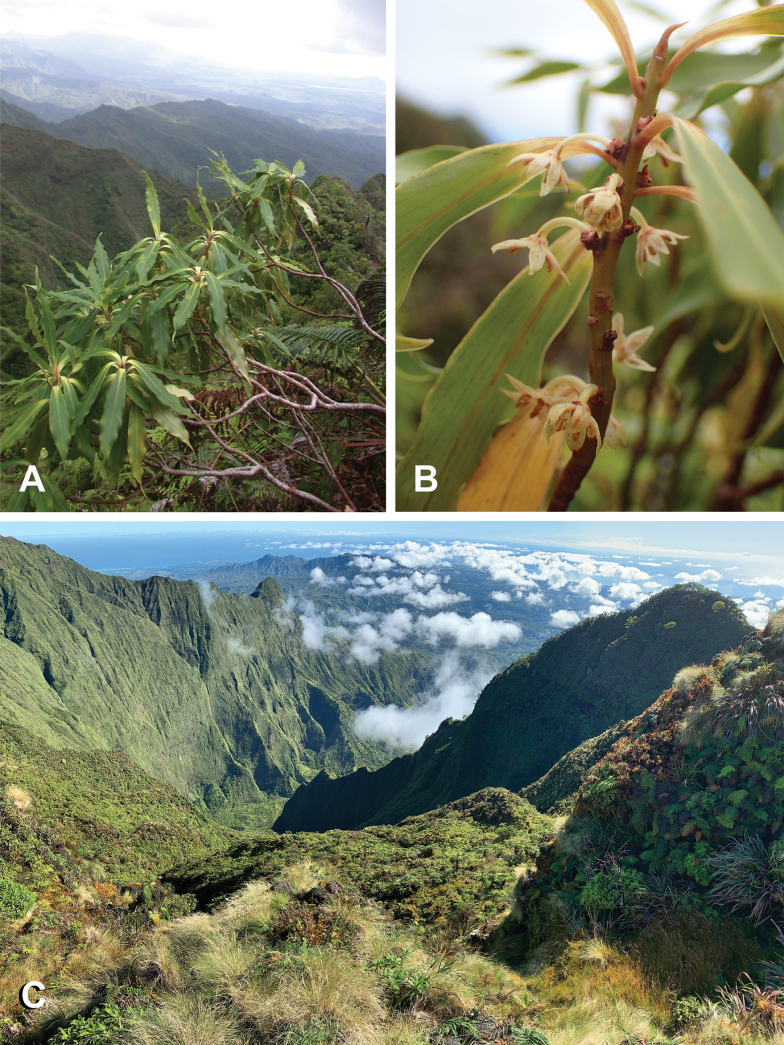
*Myrsinecirrhosa***A** habit showing leaves with characteristic undulate margins and cirrhose apex (from Kamo‘oloa headwater below Kapalaoa Kaua‘i, *Wood & Query 12824*) **B** twig with leaves and flowers (from Wai‘ahi, Kaua‘i *Wood 18139*) **C** open exposed wind-swept summit ridges of Wai‘ale‘ale, Kaua‘i representing the habitat for *Myrsinecirrhosa*. All photos by K.R. Wood.

##### Description.

Moderately branched shrubs 0.7–2 m tall; branches slender, glabrous, erect-spreading, bark brown or with orange- to reddish-brown tinge. ***Leaves*** clustered towards branch tips, blades linear-lanceolate to linear-elliptic, 4–8(–10.5) cm long, 0.9–1.5 cm wide, glabrous, adaxial surface medium green, yellow-green near base, not or sparsely black punctate, sometimes with longitudinal black streaks, abaxial surface light green, usually with several inconspicuous parallel black streaks 2.5–3.0 cm long on either side of the costa, not or scarcely black punctate, costa scarcely raised above, prominulous below, secondary veins 9–12 on each side, higher order venation conspicuously reticulate, venation prominulous on both surfaces especially below, submarginal vein present, margins entire, revolute and slightly thickened, strongly undulate in distal 2/3–3/4, apex long-acuminate, curved and hooked, base narrowly cuneate, subsessile, tapering to a winged petiole (1–)3–5 mm long. ***Flowers*** apparently perfect, 4–7 in bracteate fascicles in leaf axils or occasionally on leafless nodes, bracts broadly ovate-triangular, ca. 1.8 mm long, 1.5 mm wide, margins erose; pedicels 5–7 mm long, glabrous; calyx lobes 1.5–2.0 mm long, 0.9–1.1 mm wide, triangular-ovate, glabrous, black-streaked, margins entire; petals linear-elliptic or linear-lanceolate, 4–5 mm long, 1.3–1.5 mm wide, black-streaked, apex acute, margins slightly incurved, finely glandular ciliate towards apex; anthers 1.5–1.7 mm long, apex with slightly hooked appendage, glabrous; ovary ovoid, 1.0–1.5 mm long including the capitate stigma 0.6–0.7 mm wide. ***Drupes*** longitudinally dark streaked when immature, when ripe purple-black, globose, 7–8 mm in diameter, glabrous; pedicel 5–7 mm long.

##### Etymology.

Specific epithet refers to the curved or hooked, tendril-like leaf apices. However, the plant is shrubby and non-climbing.

##### Specimens examined

**(*paratypes*). USA, Hawaiian Islands, Kaua‘i**: Hanalei District, Wai‘ale‘ale summit area, 1524 m elev., 2 May1992, *K. R. Wood et al. 1846* (BISH, PTBG); 1524 m elev., 2 May 1992, *S. Perlman & K. R. Wood 12747* (PTBG); 1524 m elev., 28 Dec 1994, *K. R. Wood 3896* (BISH, PTBG); 1487 m elev., 29 Dec 1994, *S. Perlman et al. 14606* (PTBG, US); 1554 m elev., 30 Dec 2005, *K. R. Wood 11662* (PTBG, US); 1500 m elev., 30 Dec 2005, *K. R. Wood 11683* (BISH, PTBG); 1553 m elev., 6 Dec 2013, *A. Williams & V. Caraway AMW 27* (BISH, PTBG); Lihue District, Kamo‘oloa headwater drainage below Kapalaoa, 975 m elev., 4 Oct 1996, *K. R. Wood 5692* (PTBG); 905 m elev., 21 Feb 2008, *K. R. Wood & M. Query 12804* (BISH, PTBG); 884 m elev., 21 Feb 2008, *K. R. Wood & M. Query 12824* (BISH, PTBG); Wai‘ahi, upper central headwaters, 790 m elev., 4 Apr 2019, *K. R. Wood et al. 18139* (NY, PTBG, UC); Wai‘ahi, upper northern headwaters, 884 m elev., 25 Nov 2013, *K. R. Wood et al. 15744* (BISH, CAS, PTBG).

##### Phenology.

*Myrsinecirrhosa* has been collected with flowers from December to April, and with fruit in May and December.

##### Distribution and ecology.

**Figure 4.**
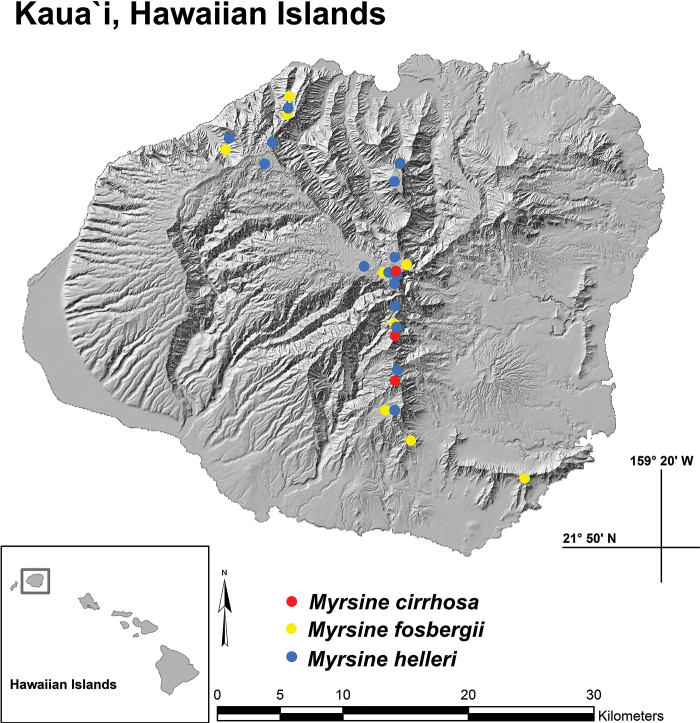
Distribution map with dots representing known locations for three *Myrsine* species on Kaua‘i, Hawaiian Islands.

*Myrsinecirrhosa* has only been documented along the central windward summit ridges and peaks of Kaua‘i, preferring lowland to predominantly montane wet ecosystems at 784–1554 m (2572–5098 ft) elevation (Fig. 4). The two plant communities where the new species has been observed include open montane bogs and also exposed windswept ridges dominated by low statured shrubs and ferns. To date, only 45 plants of *M.cirrhosa* have been documented, including ca. 20 plants within the summit bogs of Wai‘ale‘ale, renowned for being one of the wettest places on earth; ca. 20 plants in the general area of Kapalaoa peak and the very northern reaches of Wahiawa (ca. 9 km to the south of Wai‘ale‘ale); and ca. five plants found midway between those peaks along the windswept ridges of Wai‘ahi.

The open montane bog vegetation around the Wai‘ale‘ale population of *Myrsinecirrhosa* is characterised by gently contoured wet slopes dominated by a mixed composition of native sedges, grasses, herbs, shrubs and ferns. Generally, lichens and mosses are prevalent wherever pig disturbance is minimal. The low-stature vegetation (ca. < 1 m) of these open bogs is occasionally interspersed with small islands of taller (1–5 m) shrubs and trees or dissected with headwater streams of riparian vegetation bordered with forest dominated by species of *Metrosideros* Banks ex Gaertn. and *Cheirodendron* Nutt. ex Seem. In addition to the small stunted trees of *Metrosideros* and *Cheirodendron*, these remote bogs are typically composed of endemic taxa, including grass and sedge genera such as *Carex* L., *Deschampsia* P.Beauv., *Dichanthelium* (Hitchc. & Chase) Gould, *Gahnia* J.R.Forst. & G.Forst., *Machaerina* Vahl, *Oreobolus* R.Br. and *Rhynchospora* Vahl. Genera of herbs and shrubs include *Astelia* Banks & Sol. ex R.Br., *Bidens* L., *Coprosma* J.R.Forst. & G.Forst., *Drosera* L., *Dubautia* Gaudich., *Geniostoma* J.R.Forst. & G.Forst., *Geranium* Juss., *Kadua* Cham. & Schltdl., *Keysseria* Lauterb., *Melicope* J.R.Forst. & G.Forst., *Myrsine*, *Nertera* Banks ex Gaertn., *Peperomia* Ruiz & Pav., *Perrottetia* Kunth, *Plantago* L., *Stenogyne* Benth., *Vaccinium* L. and *Viola* L. Fern genera typically include *Adenophorus* Gaudich., *Asplenium* L., *Cibotium* Kaulf., *Dryopteris* Adans., *Elaphoglossum* Schott ex J.Sm., *Odontosoria* (C.Presl) Fée, *Huperzia* Bernh. and *Sadleria* Kaulf.

Exposed windswept ridges where individuals of *Myrsinecirrhosa* have been observed at the Kapalaoa, Wahiawa and Wai‘ahi sites are also dominated by endemic tree species of *Metrosideros* and *Cheirodendron* along with other shrub and tree genera, such as *Dubautia*, *Hydrangea* Gronov. ex L., *Ilex* Tourn. ex L., *Kadua*, *Leptecophylla* C.M.Weiller, *Lobelia* Plum. ex L., *Melicope*, *Polyscias* J.R.Forst. & G.Forst., *Pritchardia* Seem. & H.Wendl., *Psychotria* L., *Vaccinium*; sedges including *Machaerina*; and scrambling ferns *Dicranopteris* Bernh. and *Diplopterygium* (Diels) Nakai.

### ﻿Modification to existing key to Hawaiian *Myrsine* (Wagner et al. 1999)

To accommodate *Myrsinecirrhosa*, the following couplets can be inserted into the beginning of the existing key to Hawaiian *Myrsine* (in Wagner et al. (1999, p. 935)). Note: K = Kaua‘i; O = O‘ahu.

**Table:** 

1	Leaves linear, narrowly elliptic to narrowly lanceolate, apex attenuate to long-attenuate or long-acuminate and falcate or cirrhose-hooked	**2**
–	Leaves variable in shape, apex short-acuminate, acute to obtuse or rounded, ± emarginate	**5**
2(1)	Leaves narrowly elliptic, 8–13(–14) cm long, 1–2(–3.3) cm wide, margins plane, not undulate, apex straight or slightly hooked; flowers 4–8 per fascicle; K, O	***M.fosbergii***
–	Leaves linear, linear-elliptic to narrowly lanceolate or rarely narrowly elliptic, 1.5–9(–13) cm long, 0.25–1.4 cm wide, margins plane, slightly revolute or undulate, apex straight, falcate, slightly hooked or strongly cirrhose-hooked; flowers 1–7 per fascicle; K	**3**
3(2)	Leaves linear, 5–9 cm long, 0.25–0.4 cm wide; petals ca. 2.2–2.5 mm long	***M.linearifolia***
–	Leaves linear-lanceolate, linear-elliptic, narrowly lanceolate or rarely narrowly elliptic, 1.5–7(–13) cm long, 0.5–1.4(–1.5) cm wide; petals 2–5 mm long	**4**
4(3)	Leaves with margins strongly undulate, apex strongly cirrhose-hooked, tendril-like; flowers 4–7 per fascicle; petals 4–5 mm long	***M.cirrhosa***
–	Leaves with margins plane to slightly revolute, apex straight, falcate or slightly hooked; flowers 1–3 per fascicle; petals 3.5 mm long	**5**
5(3)	Leaves glabrous, 4–7 cm long, subsessile; pedicels 2–4 mm long; bogs	***M.helleri***
–	Leaves glabrous, except sparsely pubescent with minute rectangular, glandular hairs at the very base, especially on younger leaves, 1.5–4(–13) cm long, petioles (0–)1–3 mm long; pedicels 3–7 mm long; bogs and forest	***M.petiolata***

### ﻿Preliminary conservation assessment

According to the guidelines set by the World Conservation Union (IUCN 2012, 2022), *Myrsinecirrhosa* is classified as Critically Endangered (CR), indicating a very high risk of extinction in its natural habitat. This assessment, summarised by the IUCN alphanumeric criteria (CR B1ab(i,ii,iii,v); B2ab(i,ii,iii,v); C2a(i); D), is based on the fact that the species has a severely limited Extent of Occurrence (EOO) of only 2 km^2^, an Area of Occupancy (AOO) of approximately 1 km^2^ and population size of fewer than 50 individuals. Threats to the habitat of *Myrsinecirrhosa* include introduced non-native animals that destroy native vegetation such as pigs (*Susscrofa*), rats (*Rattus* spp.), slugs (*Meghimatiumstriatum*) and occasional goats (*Caprahircus*) and black-tailed deer (*Odocoileushemionus*). Remote island ecosystems have low resistance to non-native competitors, especially introduced animals and plants which can be devastating to native species that have evolved in their absence (Carlquist 1974; Weller et al. 2011, 2018). Invasive non-native plant species that compound habitat degradation around *M.cirrhosa* include *Andropogonvirginicus* L., *Axonopusfissifolius* (Raddi) Kuhlm., *Miconiacrenata* (Vahl) Michelang. (syn. *Clidemiahirta* (L.) D.Don), *Cyperusmeyenianus* Kunth, *Erechtitesvalerianifolius* (Link ex Spreng.) DC., *Juncusplanifolius* R.Br., *Paspalumconjugatum* P.J.Bergius, *P.urvillei* Steud., *Pterolepisglomerata* (Rottb.) Miq., *Rhodomyrtustomentosa* (Aiton) Hassk., *Rubusargutus* Link, *R.rosifolius* Sm., *Sacciolepisindica* (L.) Chase, *Setariaparviflora* (Poir.) Kerguélen, and *Sphaeropteriscooperi* (Hook. ex F.Muell.) R.M.Tryon. Landslides after heavy rains also can be a very serious threat, especially along the windswept ridge colonies of *M.cirrhosa* where a single landslide could destroy large sections of native habitat.

### ﻿Relationships and similar taxa

*Myrsinecirrhosa* most closely resembles *M.helleri*. RADseq resolved this as its closest relative (Fig. 1, Suppl. materials 1–4), but the new species can be consistently distinguished from the latter species by its strongly undulated leaf margins (Table 1, Figs 2, 3A, B). One collection was initially identified as *M.helleri* and distributed under that name (i.e. *Perlman 14606*), but it clearly represented *M.cirrhosa* on critical examination. Leaves of *M.cirrhosa* tend to be comparatively larger than those of *M.helleri* and have a combination of undulate margins and a cirrhose apex, in addition to the inflorescences having more numerous (4–7) flowers per fascicle and longer petals. Populations of *M.helleri* from Wahiawa and Namolokama may have leaves with a slightly hooked apex, but the margins are not undulate and inflorescences have fewer (1–3) flowers per fascicle. The type of *Myrsinehelleri* is from the headwaters of the Wahiawa Stream area on Kaua‘i, where it may grow sympatrically with *M.cirrhosa* (see Wagner et al. (1999) for synonymy and Wagner and Shannon (1999) for typification). *Myrsinecirrhosa* also was observed to grow sympatrically with *M.helleri* at Wai‘ale‘ale summit (*Wood & Nishek 11683*, BISH, PTBG). However, the two species remain distinct morphologically. One collection from Wai‘ale‘ale summit (*Wood 3894*, PTBG, US) is intermediate, having larger leaves with undulate margins, but lacking a cirrhose apex. It likely represents a hybrid between *M.cirrhosa* and either *M.helleri* or *M.fosbergii*, which is also sympatric here, but needs further investigation.

**Table 1.** Distinguishing characters of four Kaua‘i *Myrsine* species.

**Character**	***M.cirrhosa***	***M.helleri***	***M.fosbergii***	***M.linearifolia***
Height	0.7–2 m	2–5 m	2–4 m	2.5–8 m
Lamina length	4–8(–10.5) cm	4–7 cm	8–13(–14) cm	5–9 cm
Lamina width	0.9–1.5 cm	0.5–1.4 cm	1–2(–3.3) cm	0.25–0.4 cm
Petiole length	(1–)3–5 mm	0–4 mm	0	1–2 mm
Leaf margin	undulate	plane	plane	slightly revolute
Apex	cirrhose-hooked	straight ± curved	straight	falcate ± hooked
Flowers/fascicle	4–7	1–3	4–8	1–3
Petal length	4–5 mm	3.5 mm	2.8–3.5 mm	2–2.5 mm
Pedicel length	5–7 mm	2–4 mm	5–8 mm	1–4.2 mm

## Supplementary Material

XML Treatment for Myrsine cirrhosa

## ﻿Additional information

### Author ORCIDs

David H. Lorence https://orcid.org/0000-0002-6735-9531

Kenneth R. Wood https://orcid.org/0000-0001-6446-1154

Marc S. Appelhans https://orcid.org/0000-0003-4864-5003

Warren L. Wagner https://orcid.org/0000-0001-5012-8422

### Data availability

All of the data that support the findings of this study are available in the main text or Supplementary Information.

## Supplementary materials

Supplementary material 1 RADseq phylogeny of Hawaiian *Myrsine* based on the min8 dataset Data type tif https://binary.pensoft.net/file/1074215This dataset is made available under the Open Database License (http://opendatacommons.org/licenses/odbl/1.0/). The Open Database License (ODbL) is a license agreement intended to allow users to freely share, modify, and use this Dataset while maintaining this same freedom for others, provided that the original source and author(s) are credited.David H. Lorence, Kenneth R. Wood, Marc S. Appelhans, Warren L. Wagner

Supplementary material 2 RADseq phylogeny of Hawaiian *Myrsine* based on the min11 dataset Data type tif https://binary.pensoft.net/file/1074216This dataset is made available under the Open Database License (http://opendatacommons.org/licenses/odbl/1.0/). The Open Database License (ODbL) is a license agreement intended to allow users to freely share, modify, and use this Dataset while maintaining this same freedom for others, provided that the original source and author(s) are credited.David H. Lorence, Kenneth R. Wood, Marc S. Appelhans, Warren L. Wagner

Supplementary material 3 RADseq phylogeny of Hawaiian *Myrsine* based on the min21 dataset Data type tif https://binary.pensoft.net/file/1074217This dataset is made available under the Open Database License (http://opendatacommons.org/licenses/odbl/1.0/). The Open Database License (ODbL) is a license agreement intended to allow users to freely share, modify, and use this Dataset while maintaining this same freedom for others, provided that the original source and author(s) are credited.David H. Lorence, Kenneth R. Wood, Marc S. Appelhans, Warren L. Wagner

Supplementary material 4 RADseq phylogeny of Hawaiian *Myrsine* based on the min24 dataset Data type tif https://binary.pensoft.net/file/1074218This dataset is made available under the Open Database License (http://opendatacommons.org/licenses/odbl/1.0/). The Open Database License (ODbL) is a license agreement intended to allow users to freely share, modify, and use this Dataset while maintaining this same freedom for others, provided that the original source and author(s) are credited.David H. Lorence, Kenneth R. Wood, Marc S. Appelhans, Warren L. Wagner

Supplementary material 5 Herbarium voucher information, SRA accession numbers and sequencing statistics of specimens used for RADseq Data type xlsx https://binary.pensoft.net/file/1074219This dataset is made available under the Open Database License (http://opendatacommons.org/licenses/odbl/1.0/). The Open Database License (ODbL) is a license agreement intended to allow users to freely share, modify, and use this Dataset while maintaining this same freedom for others, provided that the original source and author(s) are credited.David H. Lorence, Kenneth R. Wood, Marc S. Appelhans, Warren L. Wagner
