# Supplementary figures and images for: ﻿ Myrsinecirrhosa (Primulaceae), a distinctive new shrub species from Kaua‘i, Hawaiian Islands
Source: PhytoKeys. 2024 Jun 19;243:47–61. doi: 10.3897/phytokeys.243.123694 (PMC11208777; doi:10.3897/phytokeys.243.123694)

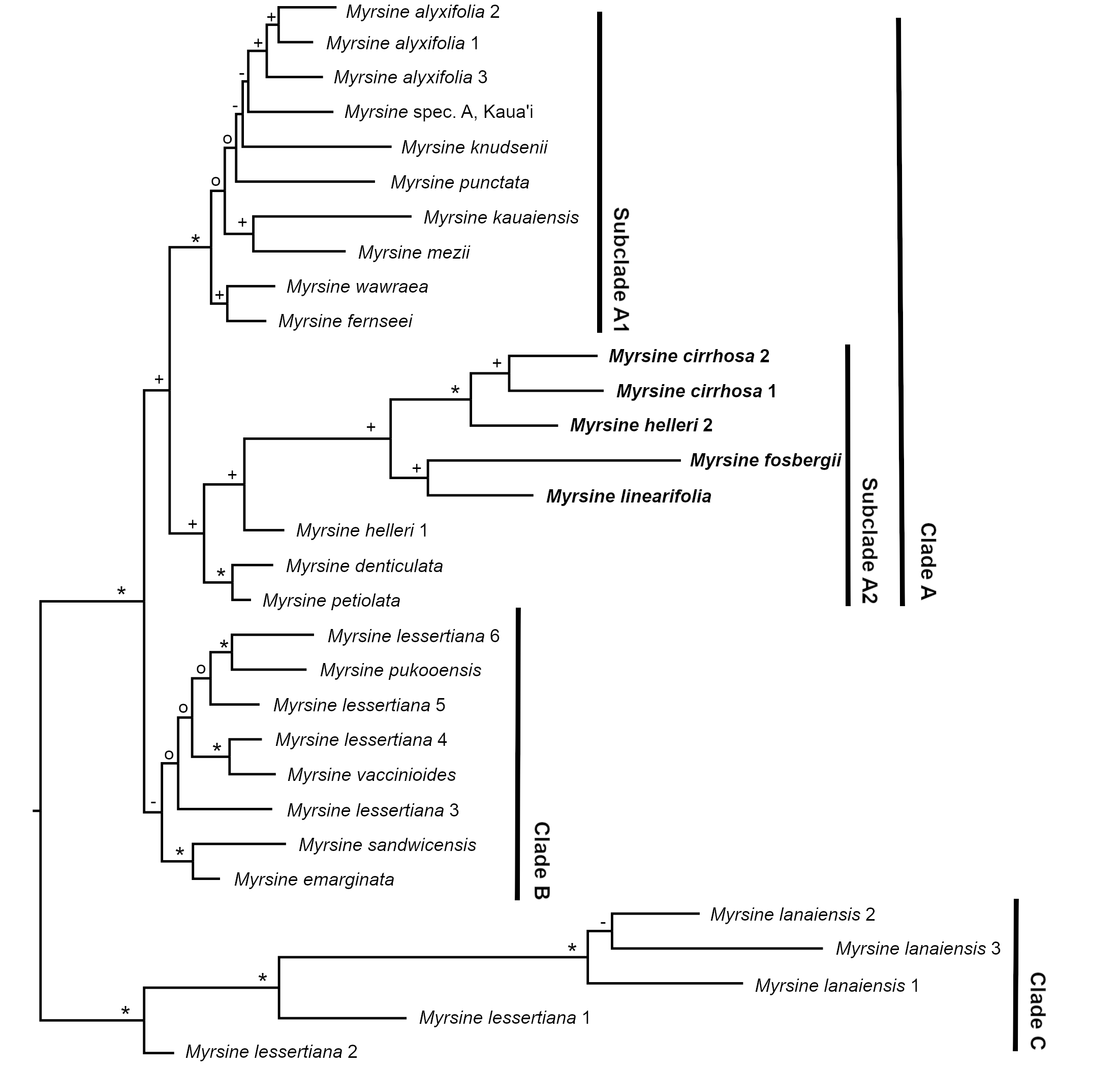

Supplement: Supplementary material 1 — RADseq phylogeny of Hawaiian Myrsine based on the min8 dataset [file phytokeys-243-047_article-123694__-s001.tif]

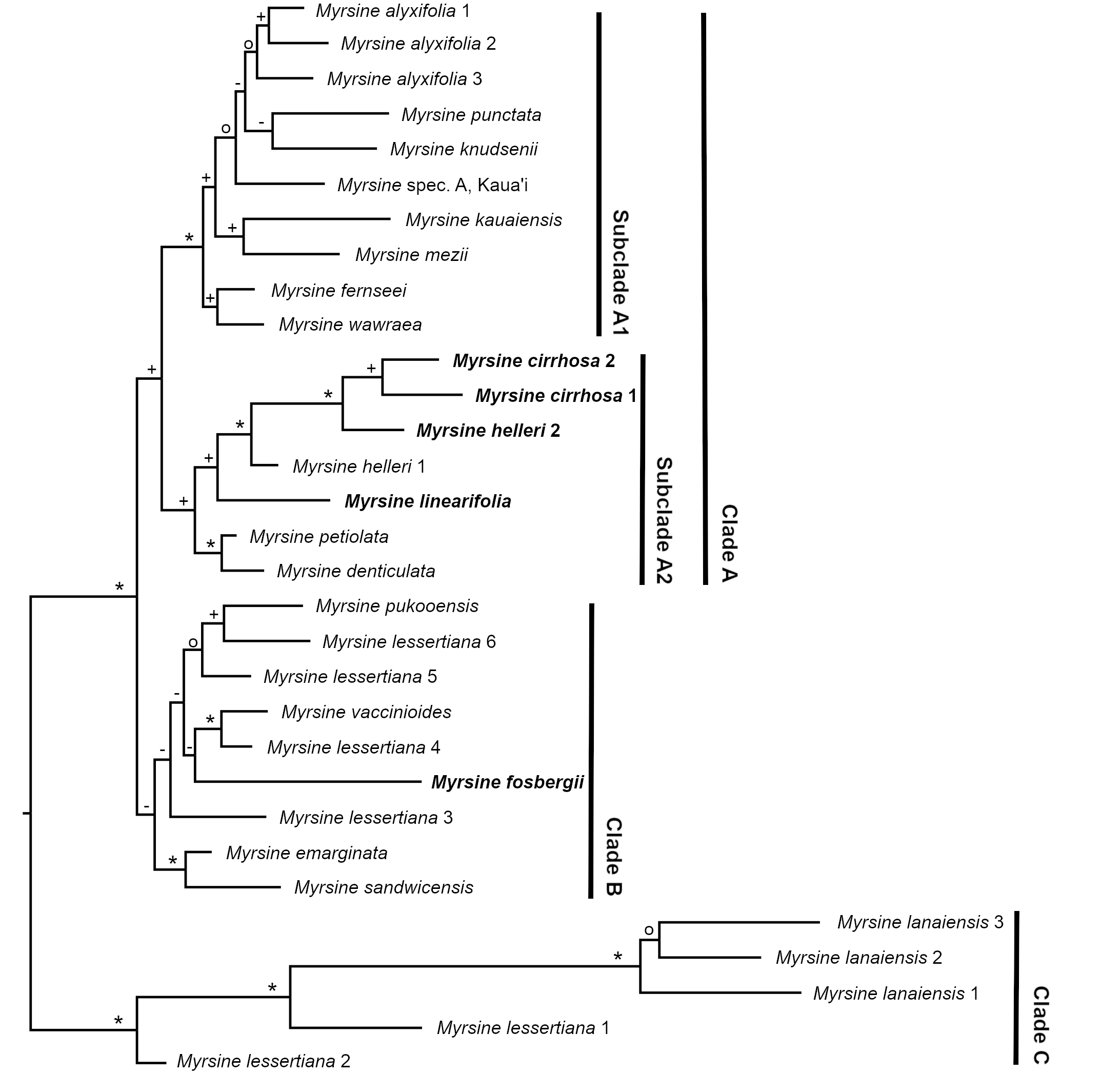

Supplement: Supplementary material 2 — RADseq phylogeny of Hawaiian Myrsine based on the min11 dataset [file phytokeys-243-047_article-123694__-s002.tif]

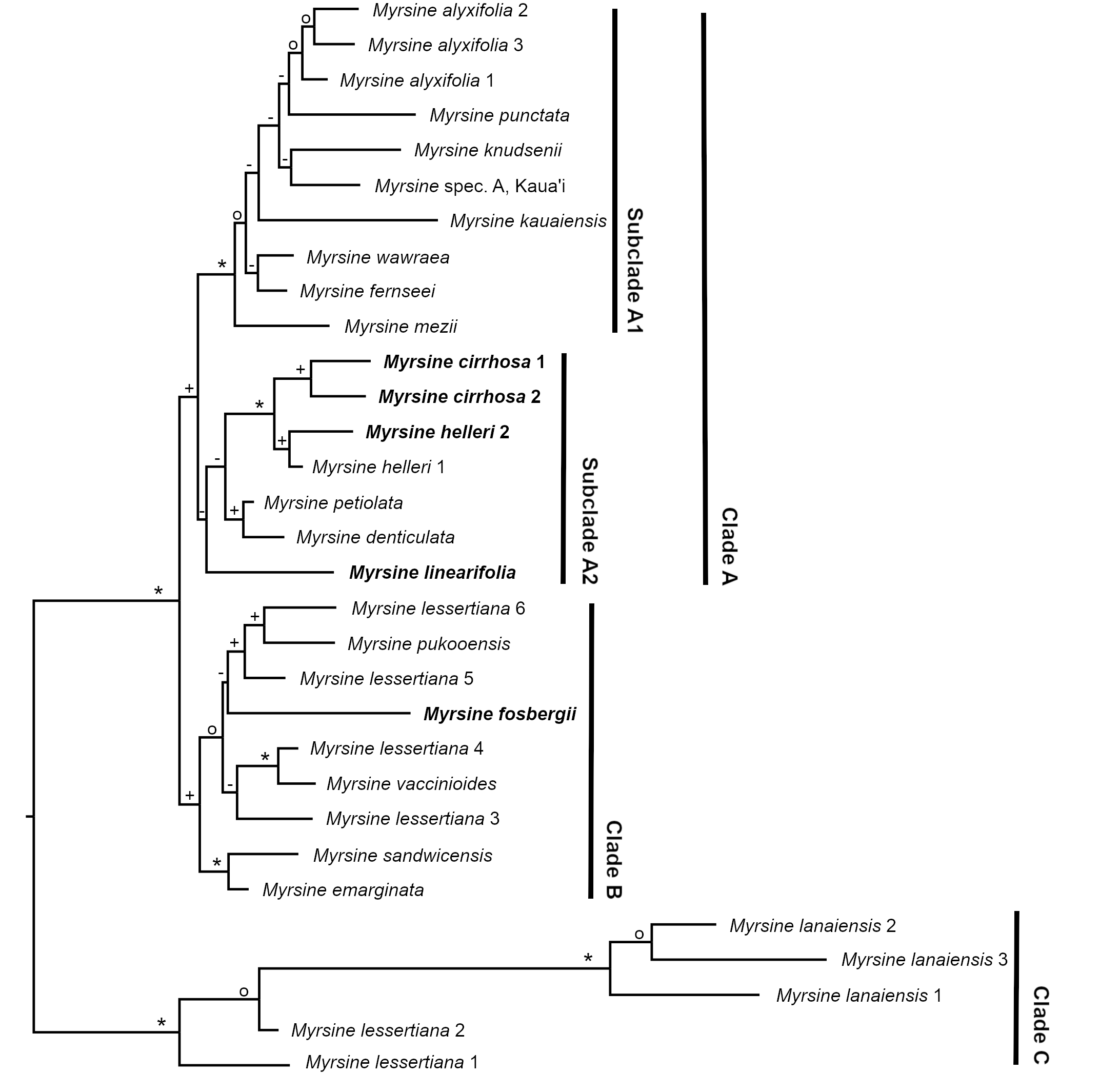

Supplement: Supplementary material 3 — RADseq phylogeny of Hawaiian Myrsine based on the min21 dataset [file phytokeys-243-047_article-123694__-s003.tif]

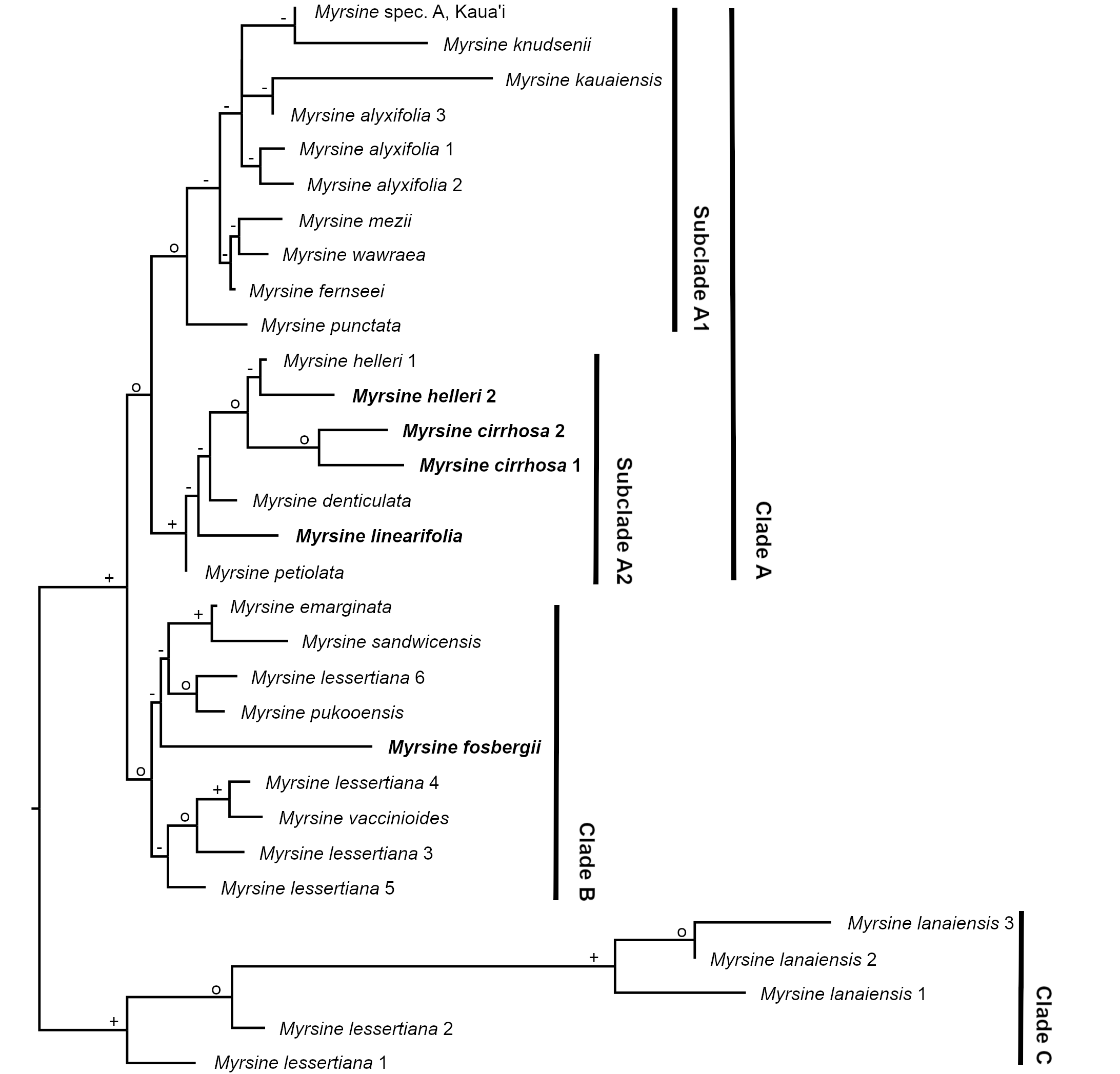

Supplement: Supplementary material 4 — RADseq phylogeny of Hawaiian Myrsine based on the min24 dataset [file phytokeys-243-047_article-123694__-s004.tif]
